# Supplementary material for: Testing the Contribution of Multi-Source Remote Sensing Features for Random Forest Classification of the Greater Amanzule Tropical Peatland
Source: Sensors (Basel). 2021 May 13;21(10):3399. doi: 10.3390/s21103399 (PMC8153014; doi:10.3390/s21103399)
Supplement: Supplementary file 1 [file sensors-21-03399-s001.zip › sensors-1188599-supplementary.pdf]

Supplementary materials: Testing the contribution of multi-source remote sensing features for random forest classification of the Greater Amanzule tropical peatland

Alex O. Amoakoh, Paul Aplin, Kwame T. Awuah, Irene Delgado-Fernandez, Cherith Moses, Carolina Peña Alonso, Stephen Kankam and Justice C. Mensah

Table S1. Confusion matrix for land cover classification of S2 dataset (original Sentinel-2 image bands).

	**Mangrove**	**Mixed swamp**	**Palm swamp**	**Bog plain**	**Natural forest**	**Sparse vegetation**	**Coconut**	**Rubber**	**Oil palm**	**Built-up**	**Bare surface**	**Water**
**Mangrove**	226	36	0	0	0	0	2	0	0	0	0	1
**Mixed swamp**	0	586	0	0	2	0	1	0	0	0	0	0
**Palm swamp**	0	1	331	0	0	0	2	0	0	0	0	0
**Bog plain**	0	0	0	264	0	0	0	0	0	4	1	0
**Natural forest**	0	1	1	0	110	0	0	3	0	0	0	0
**Sparse vegetation**	0	0	0	2	7	197	12	1	7	16	0	0
**Coconut**	1	36	5	16	37	7	171	7	0	2	0	0
**Rubber**	0	0	0	0	23	6	0	198	1	0	0	0
**Oil palm**	0	0	0	0	2	1	5	1	61	0	0	0
**Built-up**	0	0	0	13	0	2	0	0	0	227	4	12
**Bare surface**	0	0	0	4	0	1	0	0	0	10	157	0
**Water**	0	0	0	0	0	0	0	0	0	0	0	87

Table S2. Confusion matrix for land cover classification of S2+ dataset (original Sentinel-2 bands plus further spectral features – principally VIs – extracted from the Sentinel-2 bands).

	**Mangrove**	**Mixed swamp**	**Palm swamp**	**Bog plain**	**Natural forest**	**Sparse vegetation**	**Coconut**	**Rubber**	**Oil palm**	**Built-up**	**Bare surface**	**Water**
**Mangrove**	242	21	0	0	0	0	1	0	0	0	0	1
**Mixed swamp**	0	586	0	0	1	0	2	0	0	0	0	0
**Palm swamp**	0	2	330	0	0	0	1	1	0	0	0	0
**Bog plain**	0	0	1	263	0	0	0	0	0	4	0	1
**Natural forest**	0	0	1	0	111	0	1	2	0	0	0	0
**Sparse vegetation**	0	0	0	0	4	201	14	4	5	14	0	0
**Coconut**	0	29	3	12	38	4	183	9	1	3	0	0
**Rubber**	0	0	0	0	21	6	0	201	0	0	0	0
**Oil palm**	0	0	0	0	3	0	2	1	64	0	0	0
**Built-up**	0	0	0	8	0	4	2	0	0	226	0	18
**Bare surface**	0	0	0	5	0	0	0	0	0	11	156	0
**Water**	0	0	0	0	0	0	0	0	0	0	0	87

Table S3. Confusion matrix for land cover classification of S1 dataset (original Sentinel-1 image bands).

	**Mangrove**	**Mixed swamp**	**Palm swamp**	**Bog plain**	**Natural forest**	**Sparse vegetation**	**Coconut**	**Rubber**	**Oil palm**	**Built-up**	**Bare surface**	**Water**
**Mangrove**	198	24	9	1	20	0	12	1	0	0	0	0
**Mixed swamp**	38	434	9	0	90	0	11	6	0	1	0	0
**Palm swamp**	5	4	307	0	4	0	6	0	5	3	0	0
**Bog plain**	0	0	0	262	3	0	0	0	0	4	0	0
**Natural forest**	0	8	0	1	104	0	0	2	0	0	0	0
**Sparse vegetation**	0	3	0	1	140	88	1	5	0	4	0	0
**Coconut**	15	35	16	2	68	0	132	4	3	7	0	0
**Rubber**	1	11	0	0	70	0	1	145	0	0	0	0
**Oil palm**	0	0	18	0	0	0	2	0	45	5	0	0
**Built-up**	10	8	2	31	45	1	3	2	7	150	3	0
**Bare surface**	0	0	0	28	7	0	0	1	0	10	127	0
**Water**	0	0	0	10	0	0	0	0	0	0	0	77

Table S4. Confusion matrix for land cover classification of S1+ dataset (original Sentinel-1 bands plus further texture and temporal features extracted from the Sentinel-1 bands).

	**Mangrove**	**Mixed swamp**	**Palm swamp**	**Bog plain**	**Natural forest**	**Sparse vegetation**	**Coconut**	**Rubber**	**Oil palm**	**Built-up**	**Bare surface**	**Water**
**Mangrove**	192	34	1	0	28	0	3	1	0	6	0	0
**Mixed swamp**	22	502	4	0	38	0	15	5	3	0	0	0
**Palm swamp**	0	2	320	0	1	0	6	3	2	0	0	0
**Bog plain**	0	1	0	263	1	0	0	2	0	2	0	0
**Natural forest**	0	2	0	0	111	0	0	2	0	0	0	0
**Sparse vegetation**	1	15	5	5	43	109	2	57	5	0	0	0
**Coconut**	8	54	18	2	21	0	144	26	3	5	1	0
**Rubber**	3	26	1	0	21	0	2	174	0	1	0	0
**Oil palm**	0	1	9	0	2	0	1	1	55	1	0	0
**Built-up**	0	1	0	15	63	0	0	1	1	176	4	1
**Bare surface**	0	0	0	16	2	0	0	0	0	9	145	1
**Water**	0	0	0	0	3	0	0	0	0	0	0	84

Table S5. Confusion matrix for land cover classification of S2+S1+ dataset (original Sentinel-2 and Sentinel-1 bands plus further features extracted from the Sentinel-2 and Sentinel-1 bands).

	**Mangrove**	**Mixed swamp**	**Palm swamp**	**Bog plain**	**Natural forest**	**Sparse vegetation**	**Coconut**	**Rubber**	**Oil palm**	**Built-up**	**Bare surface**	**Water**
**Mangrove**	245	17	0	0	0	0	2	0	0	0	0	1
**Mixed swamp**	0	586	0	0	1	0	2	0	0	0	0	0
**Palm swamp**	0	1	332	0	0	0	1	0	0	0	0	0
**Bog plain**	0	0	1	262	0	0	0	0	0	5	1	0
**Natural forest**	0	0	0	0	113	0	0	2	0	0	0	0
**Sparse vegetation**	0	3	0	0	4	220	11	0	0	4	0	0
**Coconut**	0	36	3	4	34	5	184	10	1	5	0	0
**Rubber**	0	0	0	0	16	5	2	205	0	0	0	0
**Oil palm**	0	0	0	0	2	0	1	1	66	0	0	0
**Built-up**	0	0	0	2	0	5	1	0	0	249	0	1
**Bare surface**	0	0	0	5	0	0	0	0	0	12	155	0
**Water**	0	0	0	0	0	0	0	0	0	0	0	87

Table S6. Confusion matrix for land cover classification of S2+S1+DEM dataset (original Sentinel-2 and Sentinel-1 bands plus further features extracted from the Sentinel-2 and Sentinel-1 bands, plus STRM-derived elevation features).

	**Mangrove**	**Mixed swamp**	**Palm swamp**	**Bog plain**	**Natural forest**	**Sparse vegetation**	**Coconut**	**Rubber**	**Oil palm**	**Built-up**	**Bare surface**	**Water**
**Mangrove**	255	8	0	0	0	0	1	0	0	0	0	1
**Mixed swamp**	0	587	0	0	0	0	2	0	0	0	0	0
**Palm swamp**	0	1	332	0	0	0	1	0	0	0	0	0
**Bog plain**	0	0	0	264	0	0	0	0	0	5	0	0
**Natural forest**	0	1	0	0	114	0	0	0	0	0	0	0
**Sparse vegetation**	0	0	0	0	3	220	12	3	0	4	0	0
**Coconut**	0	38	2	5	20	3	200	9	0	5	0	0
**Rubber**	0	1	0	0	4	5	1	217	0	0	0	0
**Oil palm**	0	0	0	0	1	0	1	1	67	0	0	0
**Built-up**	0	0	0	4	0	4	2	0	0	247	0	1
**Bare surface**	0	0	0	5	0	0	0	0	0	12	155	0
**Water**	0	0	0	0	0	0	0	0	0	0	0	87

Tables S7–11. Feature importance for discriminating various land cover types based on the different datasets: (a) S2 (original Sentinel-2 image bands), (b) S2+ (original Sentinel-2 bands plus further spectral features – principally VIs – extracted from the Sentinel-2 bands), (c) S1 (original Sentinel-1 image bands), (d) S1+ (original Sentinel-1 bands plus further texture and temporal features extracted from the Sentinel-1 bands), and (e) S2+S1+ (original Sentinel-2 and Sentinel-1 bands plus further features extracted from the Sentinel-2 and Sentinel-1 bands). The five most important features are denoted with the letters a, b, c, d and e respectively. (ARVI = atmospherically resistant vegetation index, EVI = enhanced vegetation index, GNDVI = green normalized difference vegetation index, LSWI = land surface water index, MSAVI2 = modified soil-adjusted vegetation index, NBR = normalized burn ratio, NBR2 = normalized burn ratio 2, NDVI = normalized difference vegetation index, NDWI = normalized difference water index, NIR = near infrared, stdDev = standard deviation, SWIR = shortwave infrared, S2REP = Sentinel-2 red edge position index, VH = Sentinel-1 vertical-horizontal cross polarization, VV = Sentinel-1 vertical-vertical co-polarization.)

Table S7. Feature importance for discriminating various land cover types based on the S2 dataset.

	**Mangrove**	**Mixed swamp**	**Palm swamp**	**Bog plain**	**Natural forest**	**Sparse vegetation**	**Coconut**	**Rubber**	**Oil palm**	**Built-up**	**Bare surface**	**Water**
**Blue**	0.0024	0.0015	0.0153	0.0777	0.0305	0.0113	0.0077	0.0082	0.0138	0.2995e	0.2189	0.0152
**Green**	0.0499	0.0533e	0.0991d	0.1472d	0.0028	0.0925	0.0449	0.0304	0.0576	0.4022d	0.3949d	0.0154
**Red**	0.0271	0.0364	0.0714	0.2943b	0.0246	0.0868	0.0298	0.0160	0.0403	0.7645a	1.1068a	0.0899c
**Red Edge 1**	0.0552d	0.0507	0.0824	0.1247e	0.0299	0.0884	0.0483	0.0402	0.0596	0.2194	0.34904	0.0046
**Red Edge 2**	0.0386	0.0348	0.0516	0.0337	0.0460	0.0636	0.0457	0.0532	0.0573	0.0363	0.0806	0.0362
**Red Edge 3**	0.0571c	0.0556d	0.0785	0.0459	0.0836d	0.1026e	0.0776d	0.0907e	0.0977d	0.0435	0.1043	0.0616
**NIR**	0.0783b	0.0780c	0.1074c	0.0656	0.1170c	0.1429d	0.1071c	0.1238c	0.1340c	0.0584	0.1244	0.0880d
**Red Edge 4**	0.0892a	0.0917b	0.1237b	0.0786	0.1335b	0.1617c	0.1237b	0.1417b	0.1533b	0.0679	0.1286	0.1033b
**SWIR1**	0.0508e	0.1000a	0.1908a	0.3159a	0.1478a	0.2868a	0.1345a	0.2147a	0.1859a	0.4884c	0.7082b	0.1562a
**SWIR2**	0.0079	0.0489	0.0946e	0.2797c	0.0645e	0.1776b	0.0571e	0.1060d	0.0851e	0.6602b	0.6738c	0.0860e

Table S8. Feature importance for discriminating various land cover types based on the S2+ dataset.

	Mangrove	Mixed swamp	Palm swamp	Bog plain	Natural forest	Sparse vegetation	Coconut	Rubber	Oil palm	Built-up	Bare surface	Water
**Blue**	0.0016	0.0009	0.0000	0.0474	0.0188	0.0087	0.0050	0.0049	0.0087	0.1819	0.1360	0.0093
**Green**	0.0276	0.0295	0.0543	0.0811e	0.0017	0.0527	0.0248	0.0173	0.0314	0.2200e	0.2189	0.0086
**Red**	0.0144	0.0192	0.0375	0.1551a	0.0129	0.0483	0.0155	0.0087	0.0211	0.4005a	0.5858a	0.0476
**Red Edge 1**	0.0237	0.0220	0.0353	0.0538	0.0130	0.0386	0.0210	0.0174	0.0256	0.0940	0.1501	0.0019
**Red Edge 2**	0.0217	0.0198	0.0291	0.0191	0.0263	0.0366	0.0261	0.0304	0.0323	0.0205	0.0453	0.0205
**Red Edge 3**	0.0259	0.0255	0.0356	0.0210	0.0383	0.0476	0.0356	0.0417	0.0444	0.0197	0.0472	0.0280
**NIR**	0.0208	0.0210	0.0286	0.0176	0.0315	0.0391	0.0289	0.0335	0.0357	0.0155	0.0331	0.0235
**Red Edge 4**	0.0184	0.0191	0.0255	0.0163	0.0278	0.0341	0.0258	0.0296	0.0317	0.0140	0.0264	0.0214
**SWIR1**	0.0208	0.0415e	0.0785c	0.1302c	0.0613e	0.1212b	0.0556e	0.0895	0.0763e	0.2010	0.2947b	0.0643d
**SWIR2**	0.0028	0.0168	0.0322	0.0952d	0.0222	0.0622	0.0194	0.0366	0.0289	0.2247d	0.2302e	0.0293
**NDVI**	0.1052a	0.1037a	0.1089a	0.0548	0.1294b	0.1188c	0.1160b	0.1249	0.1239b	0.0101	0.0050	0.0949a
**NDVI stdDev**	0.0499d	0.0013	0.0046	0.0167	0.0023	0.0101	0.0024	0.0002	0.0021	0.0343	0.1024	0.0100
**NDWI**	0.1033b	0.1026b	0.1087b	0.0796	0.1301a	0.1249a	0.1180a	0.1283	0.1274a	0.0354	0.0722	0.0808b
**EVI**	0.0459e	0.0456d	0.0606e	0.0177	0.0747d	0.0832e	0.0643d	0.0759	0.0793d	0.0233	0.0101	0.0613e
**MSAVI2**	0.0606c	0.0598c	0.0765d	0.0204	0.0974c	0.1019d	0.0826c	0.0970	0.0999c	0.0317	0.0201	0.0774c
**LSWI**	0.0369	0.0084	0.0118	0.1487b	0.0212	0.0199	0.0162	0.0047	0.0190	0.2867b	0.2464d	0.0399
**ARVI**	0.0000	0.0000	0.0000	0.0000	0.0000	0.0000	0.0000	0.0000	0.0000	0.0000	0.0000	0.0000
**NBR**	0.0390	0.0253	0.0206	0.0673	0.0334	0.0104	0.0312	0.0238	0.0329	0.2286c	0.1300	0.0089
**NBR2**	0.0272	0.0239	0.0279	0.0130	0.0286	0.0258	0.0289	0.0292	0.0299	0.0109	0.0204	0.0166
**GNDVI**	0.0109	0.0073	0.0108	0.0767	0.0169	0.0009	0.0067	0.0069	0.0070	0.1262	0.2529c	0.0493
**S2REP**	0.0000	0.0000	0.0000	0.0000	0.0000	0.0000	0.0000	0.0000	0.0000	0.0000	0.0000	0.0000

Table S9. Feature importance for discriminating various land cover types based on the S1 dataset.

	**Mangrove**	**Mixed swamp**	**Palm swamp**	**Bog plain**	**Natural forest**	**Sparse vegetation**	**Coconut**	**Rubber**	**Oil palm**	**Built-up**	**Bare surface**	**Water**
**VH**	0.5737b	0.5775b	0.4974b	0.2387a	0.5504b	0.5014b	0.5265b	0.5259b	0.4619b	0.4497b	0.1222b	0.2300b
**VV**	0.6865a	0.6534a	0.6091a	0.1091b	0.5991a	0.5108a	0.6050a	0.5456a	0.5762a	0.5243a	0.1354a	0.4029a

Table S10. Feature importance for discriminating various land cover types based on the S1+ dataset.

	Mangrove	Mixed swamp	Palm swamp	Bog plain	Natural forest	Sparse vegetation	Coconut	Rubber	Oil palm	Built-up	Bare surface	Water
**VH**	0.2478a	0.2495a	0.2145a	0.1032a	0.2374a	0.2174a	0.2273a	0.2279a	0.1998a	0.1949c	0.0449	0.0991b
**VV**	0.1963b	0.1866b	0.1730b	0.0307b	0.1705b	0.1457b	0.1724b	0.1565b	0.1637b	0.1495e	0.0351	0.1147a
**VH stdDev**	0.1279c	0.1431c	0.0650c	0.0195c	0.1426c	0.0922c	0.0941c	0.0983c	0.0587c	0.1066	0.0480e	0.0805c
**VV stdDev**	0.0707e	0.0644e	0.0436e	0.0034	0.0619	0.0319e	0.0470e	0.0380e	0.0394e	0.1035	0.0069	0.0386e
**VV variance**	0.0109	0.0046	0.0173	0.0047	0.0556	0.0035	0.0094	0.0084	0.0127	0.2045b	0.0733d	0.0179
**VV contrast**	0.0161	0.0103	0.0232	0.0060e	0.0802e	0.0036	0.0126	0.0083	0.0171	0.3046a	0.1030c	0.0242
**VV correlation**	0.0064	0.0001	0.0054	0.0052	0.0081	0.0074	0.0054	0.0170	0.0039	0.0047	0.0115	0.0086
**VH variance**	0.0027	0.0018	0.0178	0.0003	0.0369	0.0120	0.0114	0.0001	0.0149	0.1036	0.1431b	0.0122
**VH contrast**	0.0030	0.0011	0.0270	0.0032	0.0547	0.0188	0.0179	0.0042	0.0228	0.1618d	0.1981a	0.0168
**VH correlation**	0.0064	0.0018	0.0027	0.0135d	0.0100	0.0093	0.0061	0.0192	0.0047	0.0065	0.0224	0.0085
**$VV_{\Delta amplitude}$**	0.0697	0.0624	0.0436	0.0044	0.0592	0.0314	0.0467	0.0378	0.0390	0.0949	0.0123	0.0362
**$VH_{\Delta amplitude}$**	0.1062d	0.1173d	0.0545d	0.0034	0.1144d	0.0783d	0.0780d	0.0811d	0.0488d	0.0772	0.0405	0.0656d

Table S11. Feature importance for discriminating various land cover types based on the S2+S1+ dataset.

	Mangrove	Mixed swamp	Palm swamp	Bog plain	Natural forest	Sparse vegetation	Coconut	Rubber	Oil palm	Built-up	Bare surface	Water
**Blue**	0.0016	0.0009	0.0074	0.0377	0.0146	0.0065	0.0036	0.0036	0.0069	0.1434	0.1100	0.0072
**Green**	0.0218	0.0235	0.0437e	0.0650d	0.0014	0.0413	0.0203	0.0137	0.0247	0.1758e	0.1799e	0.0067
**Red**	0.0118	0.0169	0.0332	0.1369a	0.0113	0.0432	0.0146	0.0077	0.0179	0.3518a	0.5289a	0.0415d
**Red Edge 1**	0.0216	0.0200	0.0325	0.0492	0.0118	0.0351	0.0195	0.0158	0.0231	0.0857	0.1412	0.0018
**Red Edge 2**	0.0108	0.0097	0.0144	0.0094	0.0128	0.0175	0.0129	0.0148	0.0158	0.0100	0.0230	0.0101
**Red Edge 3**	0.0044	0.0043	0.0060	0.0035	0.0064	0.0078	0.0060	0.0070	0.0075	0.0033	0.0082	0.0047
**NIR**	0.0085	0.0085	0.0117	0.0071	0.0128	0.0155	0.0118	0.0135	0.0145	0.0063	0.0140	0.0096
**Red Edge 4**	0.0058	0.0059	0.0079	0.0050	0.0085	0.0103	0.0080	0.0091	0.0098	0.0043	0.0085	0.0066
**SWIR1**	0.0095	0.0189	0.0362	0.0598e	0.0279	0.0553e	0.0260	0.0408	0.0348	0.0923	0.1376	0.0296
**SWIR2**	0.0024	0.0157	0.0305	0.0902c	0.0207	0.0595d	0.0191	0.0344	0.0269	0.2119c	0.2249b	0.0277
**NDVI**	0.0620c	0.0606c	0.0639c	0.0319	0.0756c	0.0685c	0.0679b	0.0730c	0.0727c	0.0061	0.0031	0.0556a
**NDVI stdDev**	0.0349	0.0009	0.0029	0.0119	0.0016	0.0087	0.0019	0.0000	0.0016	0.0232	0.0683	0.0069
**VH**	0.1102	0.1108a	0.0954a	0.0457	0.1056a	0.0964a	0.1009a	0.1014a	0.0887a	0.0866	0.0225	0.0441c
**VV**	0.0645a	0.0614	0.0571d	0.0102	0.0562	0.0479	0.0567d	0.0517d	0.0541d	0.0491	0.0122	0.0377
**VH stdDev**	0.0613b	0.0692b	0.0317	0.0093	0.0694d	0.0456	0.0454e	0.0480e	0.0282	0.0515	0.0210	0.0391e
**VV stdDev**	0.0362	0.0335	0.0228	0.0016	0.0324	0.0176	0.0247	0.0200	0.0205	0.0552	0.0033	0.0201
**NDWI**	0.0612d	0.0605d	0.0642b	0.0468	0.0766b	0.0728b	0.0697c	0.0756b	0.0753b	0.0207	0.0430	0.0478b
**EVI**	0.0237	0.0232	0.0311	0.0089	0.0379	0.0410	0.0330	0.0385	0.0405	0.0120	0.0049	0.0313
**MSAVI2**	0.0251	0.0244	0.0314	0.0082	0.0395	0.0403	0.0338	0.0394	0.0409e	0.0131	0.0081	0.0317
**LSWI**	0.0318	0.0072	0.0097	0.1260b	0.0178	0.0210	0.0129	0.0041	0.0164	0.2432b	0.2033c	0.0337
**ARVI**	0.0000	0.0000	0.0000	0.0000	0.0000	0.0000	0.0000	0.0000	0.0000	0.0000	0.0000	0.0000
**NBR**	0.0327	0.0210	0.0171	0.0564	0.0276	0.0060	0.0252	0.0196	0.0275	0.1903d	0.1088	0.0074
**NBR2**	0.0267	0.0232	0.0273	0.0125	0.0279	0.0245	0.0280	0.0284	0.0292	0.0107	0.0193	0.0162
**VV variance**	0.0008	0.0009	0.0037	0.0009	0.0118	0.0006	0.0020	0.0016	0.0030	0.0427	0.0162	0.0038
**VV contrast**	0.0016	0.0023	0.0054	0.0012	0.0186	0.0006	0.0029	0.0017	0.0044	0.0695	0.0252	0.0056
**VV correlation**	0.0003	0.0000	0.0004	0.0004	0.0006	0.0006	0.0004	0.0013	0.0002	0.0004	0.0009	0.0006
**VH variance**	0.0005	0.0004	0.0041	0.0000	0.0086	0.0027	0.0026	0.0002	0.0036	0.0234	0.0350	0.0028

**VH contrast**	0.0012	0.0003	0.0080	0.0007	0.0164	0.0053	0.0053	0.0015	0.0070	0.0467	0.0616	0.0050
**VH correlation**	0.0003	0.0001	0.0001	0.0008	0.0006	0.0006	0.0004	0.0011	0.0002	0.0004	0.0014	0.0005
**GNDVI**	0.0084	0.0052	0.0079	0.0550	0.0120	0.0018	0.0044	0.0048	0.0053	0.0899	0.1826d	0.0352
**$VV_{\Delta amplitude}$**	0.0311	0.0280	0.0197	0.0018	0.0268	0.0152	0.0211	0.0172	0.0177	0.0437	0.0054	0.0164
**$VH_{\Delta amplitude}$**	0.0490e	0.0546e	0.0256	0.0013	0.0537e	0.0361	0.0362	0.0380	0.0225	0.0361	0.0177	0.0306
**S2REP**	0.0000	0.0000	0.0000	0.0000	0.0000	0.0000	0.0000	0.0000	0.0000	0.0000	0.0000	0.0000
